# Antitumor activity and antioxidant status of *Streblus asper* bark against Dalton's ascitic lymphoma in mice

**DOI:** 10.1515/intox-2015-0019

**Published:** 2015-09

**Authors:** R.B. Suresh Kumar, Biswakanth Kar, Narayan Dolai, Indrajit Karmakar, Sanjib Bhattacharya, Pallab K. Haldar

**Affiliations:** 1 Department of Pharmaceutical Technology, Jadavpur University, Kolkata 700032, West Bengal India; 2 Faculty of Pharmaceutical Sciences, PCTE Group of Institutes, Ludhiana 142021, Punjab, India

**Keywords:** *Streblus asper*, Dalton's ascitic lymphoma (DAL), antitumor, antioxidant

## Abstract

*Streblus asper* Lour (Moraceae), commonly known as Siamee Rough Brush in English is widely distributed in subtropical Asia and traditionally used for several medicinal purposes. In the present study, the ethyl acetate fraction of the methanol extract from *Streblus asper* bark (EASA) was evaluated for antitumor effect against Dalton's ascitic lymphoma (DAL) in Swiss albino mice. Twenty-four hours after intraperitoneal inoculation of DAL cells in mice, EASA was administered intraperitoneally at 200 and 400 mg/kg body weight for 9 consecutive days. On the 10th day, half of the mice were sacrificed to determine the tumor growth parameters, and the rest were kept alive for survival assessment. Hematological, serum biochemical and tissue (liver, kidney) antioxidant profiles were also determined. EASA exhibited significant and dose dependent decrease in tumor growth parameters and increased survival of DAL bearing animals. EASA significantly and dose-dependently normalized the altered hematological, serum biochemical and tissue antioxidant parameters as compared with the DAL control mice. From the present study it may be concluded that S. *asper* bark possesses remarkable antitumor efficacy mediated by amelioration of oxidative stress by multiple mechanisms.

## Introduction

Cancer is considered one of the deadliest causes of morbidity and mortality all over the world. Although the disease has often been regarded principally as a problem of the developed world, more than half of all cancers occur in the developing countries (Stewart & Kleihues, 2003). Unfortunately, current available cancer chemotherapeutic agents insidiously affect the host cells, especially bone marrow, epithelial tissues, the reticuloendothelial system and gonads (Mascarenhas, 1994). The approach for minimizing unwanted toxicity is to employ newer natural products that may act by different and distinct mechanism(s) and/or precipitate less serious side effects. A number of plant or other natural product extracts have been studied for anticancer activity leading to the development of several clinically useful anticancer agents (da-Rocha *et al*., 2001). Several natural products have been contemplated to be of exceptional value in the development of effective anticancer drugs with minimum host cell toxicity.

*Streblus asper* Lour (Moraceae), commonly known as Siamee Rough Brush in English, *Sakhotaka* in Ayurveda and *Piraayan* in Siddha system of medicine is widely distributed in subtropical Asia. All parts of this plant have been traditionally used in India for several medicinal purposes (Khare, 2007). Its roots are used for ulcer, sinuses, antidote for snake bite, epilepsy and obesity; the stem is used for toothache; stem bark is used in fever, dysentery, diarrhea, stomach ache, urinary tract complaints, piles, edema and wounds. Leaves are used for eye complications, seeds in epistaxis and diarrhea. According to Ayurveda, the traditional system of Indian medicine, its stem bark is used in treatment of hemorrhage, tubercular adenitis, cervical lymphadenitis and piles (Rastogi & Dhawan, 1990; Rastogi *et al*., 2009; Caldecott, 2011). As there are no experimental reports on antitumor activity of *S. asper* bark, we found it necessary to evaluate the ethyl acetate fraction of defatted methanol extract of *S. asper* stem bark (EASA) for possible antitumor effect and influence on antioxidant status against Dalton's ascitic lymphoma (DAL) in Swiss albino mice.

## Materials and methods

### Plant material

The stem bark of *Streblus asper* (Moraceae) was collected from West Bengal, India, during March 2010 and identified at the Central National Herbarium, Botanical Survey of India, Shibpur Howrah, West Bengal, India and a voucher specimen (no. CNH/I-I/(29/2010/Tech.II) has been preserved at our laboratory for future reference. Just after collection, the plant material was washed thoroughly with running tap water, shade dried at room temperature (24-26 °C) and ground mechanically into a coarse powder.

### Drugs and chemicals

Bovine serum albumin and 5-fluorouracil from Sigma Chemical Co., St. Louis, Mo, USA; trichloroacetic acid (TCA) and 1-dichloro-2,4-dinitrobenezene (CDNB) from Merck Ltd., Mumbai, India; thiobarbituric acid (TBA), nitroblue tetrazolium chloride (NBT) from Loba Chemie, Mumbai, India; 5,5'-dithio *bis*-2-nitro benzoic acid (DTNB), phenazonium methosulphate (PMS), nicotinamide adenine dinucleotide (NADH) and reduced glutathione (GSH) from SISCO Research Laboratory, Mumbai, India. All the other reagents used were of analytical reagent grade obtained commercially. Doubled distilled water from all-glass still was employed throughout the study.

### Preparation of extract

The powdered plant material (500 g) was successively extracted with petroleum ether (60-80 °C), followed by methanol using a soxhlet extraction apparatus. The methanol extract was completely dried under reduced pressure (0.8 MPa) to get defatted methanol extract (yield: 19% w/w). The defatted methanol extract was fractioned with ethyl acetate to yield the ethyl acetate fraction (EASA, yield: 3% w/w) which was used in the present study.

### Experimental animals

Adult male Swiss albino mice weighing 20±2 g were obtained from Laboratory Animal Centre, Department of Pharmaceutical Technology, Jadavpur University, Kolkata, India. The mice were grouped and housed in polyacrylic cages (38×23×10 cm) with not more than four animals per cage and maintained under standard laboratory conditions (temperature 25±2 °C with dark/light cycle 12/12 h). They were allowed free access to standard dry pellet diet (Hindustan Lever, Kolkata, India) and water *ad libitum*. The mice were acclimatized to laboratory conditions for 10 days before commencement of the experiment. All procedures described were reviewed and approved by the University Animal Ethical Committee, Jadavpur University (Reg. no. 367001/C/CPCSEA).

### Preparation of tumor cells

Dalton's ascitic lymphoma cells (DAL) were obtained from Chittaranjan National Cancer Institute (CNCI), Kolkata, India. The DAL cells were maintained *in vivo* in Swiss albino mice by sequential intraperitoneal transplantation of 2×10^6^ cells per mice after every 10 days and used for *in vivo* study (Haldar *et al*., 2010). Before experiment, the DAL cells were collected from the donor mouse and were suspended in sterile isotonic saline and the tumor cell count was adjusted to 2×10^7^ cells/mL by sterile isotonic saline.

### Treatment schedule

The animals were divided into five groups (n=12). Except the first group, all groups received 0.1 ml of DAL cell suspension (2×10^6^ cells/mouse, i.p.). This was taken as day ‘0'. The first group served as normal saline control (received isotonic saline, 5 mL/kg body weight, i.p.). The second group served as DAL control. After 24 h of tumor inoculation the third and fourth group received EASA at the respective doses of 200 and 400 mg/kg body weight, i.p. for 9 consecutive days. Twenty-four hours after the last dose and after 18 h of fasting, blood was collected from six mice of each group by cardiac puncture for determination of hematological parameters and then they were sacrificed by cervical dislocation for the study of antitumor and tissue antioxidant parameters. The remaining six mice of each group were kept alive with food and water *ad libitum* to assess the survival parameters of the tumor bearing animals (Haldar *et al*., 2010).

### Tumor weight

The tumor weight was measured by the difference in the weight of mice before and after collection of ascitic fluid from the peritoneal cavity.

### Tumor volume

The tumor volume was measured by taking the ascitic fluid in a standard and clean graduated centrifuge tube.

### Tumor cell counts

The ascitic fluid was taken in a WBC pipette and diluted 100 times. Then one drop of the diluted suspension was placed on the Neubauer counting chamber and the numbers of cells in the 64 small squares were counted. The cells were then stained with trypan blue (0.4 % in isotonic saline) dye (trypan blue dye exclusion assay). The cells that did not take up the dye were viable and those which took the dye were non-viable. These viable and non-viable cells were counted as per following formula:

Cell count = (No. of cells × dilution factor)/(area × thickness of liquid film).

### Percentage increase in life span

The survival was monitored by recording median survival time (MST) and percentage increase in life span (%ILS) as per the following formulae:

Median survival time (MST)* = [First death + Last death]/2

*Time denoted by number of days.

%ILS = [(MST of EASA treated group/ MST of DAL control group) - 1)] × 100

### Estimation of hematological parameters

Collected blood was used for the estimation of hemoglobin (Hb) content; red blood cell (RBC) count and total white blood cell (WBC) counts (D'Armour *et al*., 1965; Wintrobe *et al*., 1961).

### Estimation of serum biochemical parameters

Collected blood was centrifuged and serum was used for the estimation of serum glutamate oxaloacetate transaminase (SGOT), serum glutamate pyruvate transaminase (SGPT), and alkaline phosphatase (ALP) by using commercially available reagent kits (Span Diagnostics Ltd., Surat, India). Serum total protein content was assessed by the method of Lowry *et al*. (1951).

### Determination of tissue antioxidant parameters

The antioxidant parameters of processed liver and kidney tissues were assessed by measuring the level of lipid peroxidation (Ohkawa *et al*., 1979), the amount of reduced glutathione (Ellman, 1959), and the enzymatic activities of catalase (Sinha, 1972) and superoxide dismutase (Kakkar *et al*., 1984). The total protein content of liver and kidney tissue for these tissue biochemical parameters was determined by the method of Lowry *et al*. (1951).

### Statistical analysis

All data were expressed as the mean ± standard error of mean (SEM). The results were analyzed for statistical significance by one-way ANOVA followed by Dunnett's multiple comparison test of significance, *p*<0.01 was considered statistically significant.

## Results

**Figure 1:**
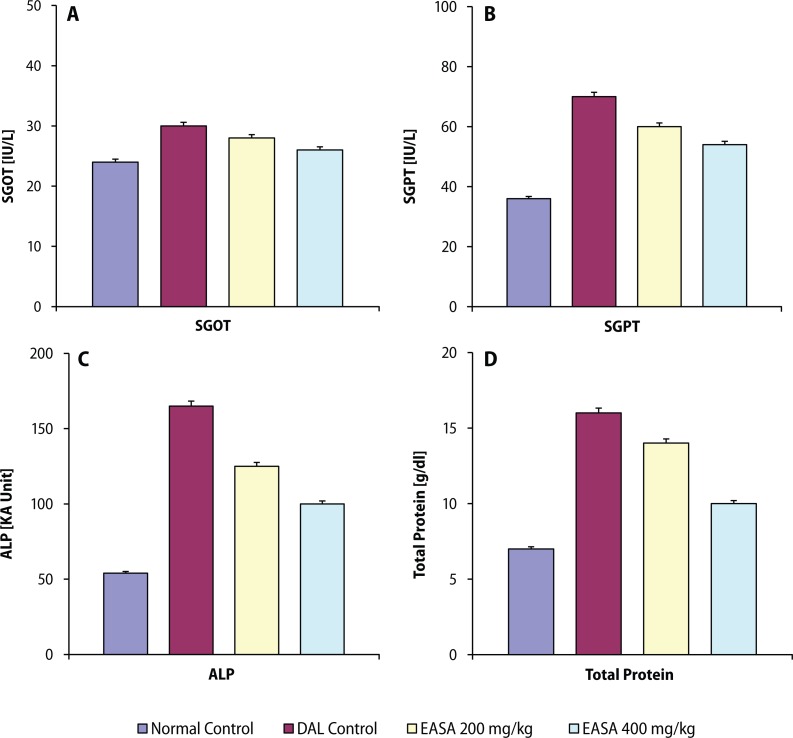
Effect of EASA on serum biochemical parameters like SGOT (A), SGPT (B), ALP (C) and total protein (D) in DAL bearing mice. Values are represented as mean ± SEM (n=6). a: DAL control group vs. normal control group, ^#^*p*< 0.01; b: All treated groups vs. DAL control group, *p<0.01.

**Figure 2:**
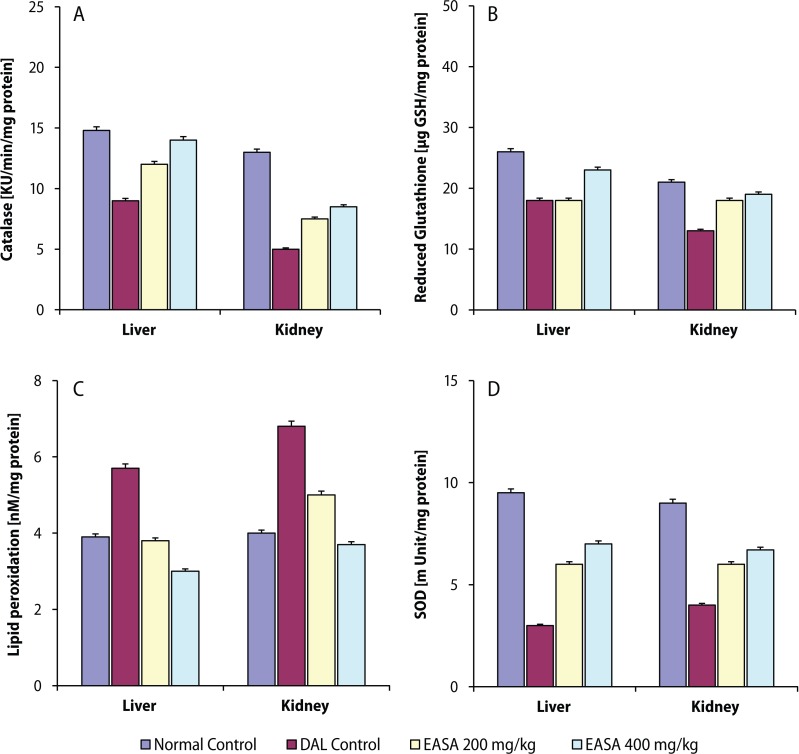
Effect of EASA on tissue antioxidant parameters like catalase (A), reduced glutathione (B), lipid peroxidation (C) and superoxide dismutase (D) in DLA bearing mice. Values are represented as mean ± SEM (n=6). a: DAL control group vs. normal control group, ^#^*p*<0.01; b: All treated groups vs. DAL control group, * *p*<0.01.

In the present study, EASA at the dose of 200 and 400 mg/kg body weight significantly increased in a dosedependent manner the survival parameters (MST, %ILS) and non-viable cell count, decreased the tumor volume and viable cell count of the tumor bearing mice as compared to those of DAL control mice (Table 1). All the altered hematological parameters were significantly and dose-dependently restored by EASA (Table 1). EASA significantly and dose-dependently brought the elevated serum biochemical parameters towards the normal levels (Figure 1). Treatment with EASA also significantly recuperated all the perturbed hepatic and renal antioxidant parameters in tumor bearing mice in a dose related way (Figure 2).

**Table 1:** Effect of EASA on tumor volume, tumor weight, viable and non-viable cell counts, median survival time (MST), percentage increase life span (% ILS) and hematological parameters (RBC, WBC and hemoglobin) in DAL bearing mice.

Parameters	Normal saline (5 ml/kg)	DAL control (2×10^6^ cell/mouse)	DAL +EASA (200 mg/kg)	DLA+EASA (400 mg/kg)
Tumor volume (ml)	–	3.9±0.02	3.38±0.25 ^b,^*	2.3±0.10 ^b,^*
Tumor weight (g)	–	3.6±0.2	3.01±0.04 ^b,^*	2.9±0.04 ^b,^*
Viable cell (x10^6^ cells/ml)	–	7.3±0.1	5.9±0.61 ^b,^*	5.3±0.41 ^b,^*
Non-viable cell(x10^6^ cells/ml)	–	0.6±0.14	3.18±0.03 ^b,^*	3.9±0.04 ^b,^*
MST (days)	–	20	24	27 ^b,^*
%ILS	–	–	20	35 ^b,^*
RBC (cells x10^6^/mm^3^)	6.60±0.12	2.15±0.05 ^a,^*	3.3±0.19 ^b,^*	4.4±0.2 ^b,^*
WBC (cells x10^3^/mm^3^)	5.30±0.17	13.1 ±0.4 ^a,^*	12.25±0.3 ^b,^*	10.6±0.39 ^b,^*
Hemoglobin(g/dl)	13.2±0.15	5.55±0.5 ^a,^*	6.43±0.14 ^b,^*	8.33±0.47 ^b,^*

Values are represented as mean ± SEM (n=6).

a Where DAL control group *vs*. normal control group, #*p*< 0.01

b All treated groups *vs*. DAL control group

* *p*<0.01.

## Discussion

The aim of the present work was to study the antitumor activity of the ethyl acetate fraction of defatted methanol extract of *S. asper* stem bark (EASA) in DAL bearing mice. The results of this study revealed that EASA at the doses of 200 and 400 mg/kg demonstrated a marked antitumor effect as evidenced by significant reduction in tumor volume, tumor weight, viable tumor cell count and increase in non-viable tumor cell count, median survival time and life span of tumor bearing hosts. EASA significantly restored the altered hematological parameters towards normal values. EASA also significantly modulated the hepatic and renal antioxidant parameters, *i.e*. lipid peroxidation, reduced glutathione level (GSH), activities of superoxide dismutase (SOD) and catalase (CAT) in tumor bearing mice.

Cancer is a pathological state involving uncontrolled proliferation of tumor cells (Evans, 2002). The increase in ascitic volume was accompanied by an increase in total cell count. The results of Trypan blue dye exclusion assay demonstrated that EASA treatment resulted in decreased viable tumor cell count with increased non-viable cell count. This indicates a direct relationship of EASA with tumor cell proliferation, implying direct cytotoxic effect of EASA on DAL cells.

The reliable criterion for judging the value of any anticancer drug is prolongation of life span of the tumor bearing animal (Clarkson & Burchneal, 1965). A ≥ 25 % increase in life span of tumor bearing animals is considered to be indicative of significant antitumor activity (Andreani & Galatulas, 1983). In the present study, the reduced tumor volume and extended survival time of EASA treated tumor bearing mice is suggestive of the delaying impact of EASA on tumor proliferation.

In cancer chemotherapy, the major problems encountered are myelosuppression and anemia due to reduction in erythrocytes and hemoglobin content and increase in leukocytes (Price & Greenfield, 1958; Hogland, 1982). EASA dose-dependently and significantly increased the erythrocyte count and hemoglobin contents and decreased the leukocyte count as compared to those of DAL control. These parameters indicate that EASA exerted a less toxic effect on the blood and hematopoietic system, thereby maintaining the normal hematological profile in tumor bearing mice.

Serum biochemical parameters like SGOT, SGPT, ALP and total protein are known as hepatic function parameters, elevation of which occurs due to impaired hepatic functions in tumor bearing mice (Haldar *et al*., 2010). Decrease of the elevated serum biochemical parameters towards the normal levels by EASA treatment indicated that the hepatic functions could be maintained normal in DAL bearing mice.

Neoplastic growth has been found to co-exist with impairment in the endogenous antioxidant status. Oxidative stress is caused by a relative overproduction of oxidative free radicals that result in lipid peroxidation leading to degradation of cellular macromolecules (Oberley, 2002). Reactive oxygen species (ROS) result in lipid peroxidation and subsequent increase of the levels of malondialdehyde (MDA) and other thiobarbituric acid reactive substances (TBARS) which lead to degradation of cellular macromolecules (Yoshikawa *et al*., 1983). A marked increase in the concentration of TBARS in DAL control mice indicated enhanced lipid peroxidation leading to tissue injury and failure of the endogenous antioxidant defense mechanisms to prevent overproduction of ROS. Decrease in lipid peroxidation by EASA implies that its antitumor action may be mediated by preventing the overproduction of oxidative free radicals.

Glutathione, a potent inhibitor of the neoplastic process, plays an important role in the endogenous nonenzymatic antioxidant system. In its reduced form (GSH) it acts as reducing agent and detoxifies hydrogen peroxide (Bhattacharya *et al*., 2011). Besides its involvement in the detoxification process, GSH probably also plays an important role in lymphocyte function and depletion in GSH content associated with impaired immune response and increased risk of malignancy (Gmunder & Droge, 1991). The depleted reduced glutathione (GSH) may be due to reduction in GSH synthesis or degradation of GSH by oxidative stress in DAL bearing animals. EASA treatment significantly elevated the reduced hepatic and renal GSH contents towards normal in tumor bearing mice. The results exhibited that the antitumor activity of EASA was accompanied with enhancement of cellular non-enzymatic antioxidant defense, by which EASA may have exerted its antitumor role.

The enzymatic antioxidant mechanisms are involved in the protection of tissues from oxidative stress by eliminating free radicals. Superoxide dismutase (SOD) and catalase (CAT) are involved in the clearance of superoxide and hydrogen peroxide radicals (Oberley & Oberley, 1997). Elevation of enzyme activities like SOD and CAT in EASA treated tumor bearing mice revealed the activation of enzymatic antioxidant defense mechanisms by which EASA resulted in amelioration of DAL-induced oxidative stress. Thus EASA treatment resulted in amelioration of tumor-induced oxidative stress by multiple mechanisms, involving modulation of lipid peroxidation, endogenous non-enzymatic (GSH) and enzymatic (SOD, CAT) antioxidant systems.

In the present investigation, EASA treatment was found to significantly reduce tumor proliferation, viability of tumor cells, to normalize the hematological and serum biochemical profiles, and to extend the survival time (life span), as compared with DAL control mice. EASA treatment resulted also in significant amelioration of tumor-induced oxidative stress by multiple mechanisms, involving modulation of lipid peroxidation, endogenous non-enzymatic (GSH) and enzymatic (SOD, CAT) antioxidant and detoxification systems. From the present study it can be concluded that the ethyl acetate fraction of defatted methanol extract from *S. asper* bark demonstrated remarkable antitumor efficacy against Dalton's ascitic lymphoma in Swiss albino mice, mediated plausibly by virtue of ameliorating oxidative stress by augmenting the endogenous antioxidant status.
